# Emergent spectral properties of river network topology: an optimal channel network approach

**DOI:** 10.1038/s41598-017-11579-1

**Published:** 2017-09-13

**Authors:** Armaghan Abed-Elmdoust, Arvind Singh, Zong-Liang Yang

**Affiliations:** 1 University of Texas at Austin, Department of Geological Sciences, Jackson School of Geosciences, Austin, 78712 USA; 2 University of Central Florida, Department of Civil, Environmental and Construction Engineering, Orlando, 32816 USA

## Abstract

Characterization of river drainage networks has been a subject of research for many years. However, most previous studies have been limited to quantities which are loosely connected to the topological properties of these networks. In this work, through a graph-theoretic formulation of drainage river networks, we investigate the eigenvalue spectra of their adjacency matrix. First, we introduce a graph theory model for river networks and explore the properties of the network through its adjacency matrix. Next, we show that the eigenvalue spectra of such complex networks follow distinct patterns and exhibit striking features including a spectral gap in which no eigenvalue exists as well as a finite number of zero eigenvalues. We show that such spectral features are closely related to the branching topology of the associated river networks. In this regard, we find an empirical relation for the spectral gap and nullity in terms of the energy dissipation exponent of the drainage networks. In addition, the eigenvalue distribution is found to follow a finite-width probability density function with certain skewness which is related to the drainage pattern. Our results are based on optimal channel network simulations and validated through examples obtained from physical experiments on landscape evolution. These results suggest the potential of the spectral graph techniques in characterizing and modeling river networks.

## Introduction

River networks have been a subject of research for many years. They are central to several processes occurring on river ecosystem and provide primary pathways to transport environmental fluxes such as water, nutrient, and sediment^1–5^. Understanding and quantifying their structure and dynamics is essential for both advancing fundamental knowledge about their emergence and evolution as well as for management and prediction of environmental processes and fluxes operating upon them^1–9^.

Consisting of branching channels, river networks’ display highly nonlinear dynamics and complex topology. They have been shown to exhibit various properties such as self-similarity and scaling laws across a range of scales commonly observed in complex (both natural and engineered) networks^10–13^. Ranging from seminal works of Horton^14, 15^ and Shreve^16–18^ which set the foundation of stream ordering schemes, several aspects related to river network geomorphology and topology have been explored using physical, theoretical, numerical and field approaches. However, studies that specifically relate geometric and topologic properties of river network are still lacking.

Along different lines, spectral graph theory has a long history^19, 20^ and is a rapidly growing field in connection with complex networks^13^. Predominantly, spectral graph theory deals with the study of graphs through the eigenvalues and eigenvectors of their associated matrices. Because of the generality of problems involving graphs, spectral graph techniques are deeply connected with different fields of science and engineering ranging from quantum chemistry^21^ and communication networks^22^ to computer science^23^ and combinatorics^24^ to mention a few.

Considering the importance of the spectral graph techniques in all such areas and given recent advances in complex network characterization, of interest would be to explore the ramifications of these theories and techniques in one of the most interesting examples of naturally occurring complex networks; river drainage networks. Note that graph theory has been previously used to study drainage network topology^25^. More recently, the topologic and dynamic complexity of delta channel networks have been investigated through a graph-theoretic approach^26, 27^. However, to the best of our knowledge, the spectral properties of river network topology, such as eigenvalue distribution and spectral gap, have never been studied.

Using a graph theoretic formulation, here we investigate the eigenvalue spectrum of the adjacency matrix of river networks. First, we consider drainage networks generated on two-dimensional lattices through an optimal channel network (OCN) model. We then discuss general properties of the adjacency matrix and the eigenvalue spectrum associated with such networks. The main characteristics of the eigenvalue spectrum are extracted and their relation with the topology of the river network is discussed. The statistical behavior of the eigenvalues is also investigated and is compared with that of well-known networks. Finally, we explore examples from physical experiments on landscape evolution and show that our results are applicable to a variety of complex river networks formed under different external forcings.

## Results

### Adjacency matrix and properties of river networks

In general, the flow paths in a river network can be described through a directed graph which can itself be represented by an *N* × *N* adjacency matrix *A*, where *N* is the number of nodes. Adjacency matrix, *A* can be expressed as,

1 $${a}_{ij}=\{\begin{array}{ll}1: & i\,{\rm{flows}}\,{\rm{to}}\,j;\\ 0: & {\rm{otherwise}}\end{array}$$

and characterizes the connection between two adjacent nodes, along the flow direction. Thus, the structure of a river network can be fully determined through the coordinates of its nodes and its adjacency matrix which respectively describe the geometry and the topology of the network. In other words, a river network can be considered as a spanning tree on a two-dimensional regularly spaced square lattice grid of nodes *N* which can, in principle, be surrounded by an arbitrary shaped boundary describing the shape of a river basin^28^. Each node on this grid can only be connected to its eight nearest neighbors through a link. The connecting links, are directed links representing the flow direction. Although each node can have an inflow from multiple upstream nodes, it can only have one outflow to the downstream node; thus each link is uniquely associated with its upstream node. By considering an exception from this rule, here we introduce an outlet node (associated with the outlet of a river) which does not have any downstream node.

Following equation (1), one can directly list the following properties for the asymmetric adjacency matrix *A*: (i) given that there is only one node downstream of each node, each row of the adjacency matrix *A* includes only 1 and the only exception is the row associated to the outlet node which does not have any downstream node therefore this row is completely zero. (ii) Number of 1’s in each column corresponds to the number of nodes directly upstream of the corresponding node. (iii) All the diagonal elements of *A* are zero.

**Figure 1:**
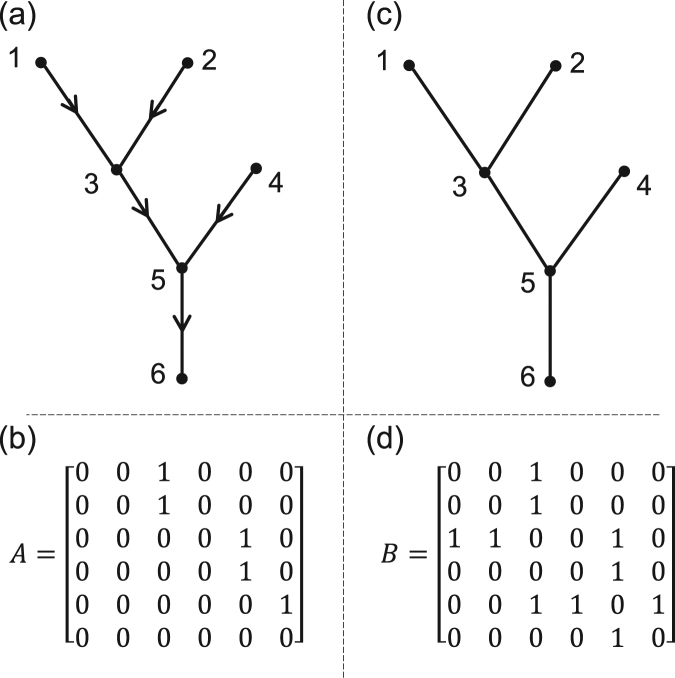
An exemplary stream network and its adjacency matrix. (**a**) The directed graph representation, (**b**) the asymmetric adjacency matrix *A*, (**c**) the undirected graph representation, and (**d**) the symmetric adjacency matrix *B*.

Figure 1 shows an exemplary river basin (Fig. 1a) along with its adjacency matrix (Fig. 1b) revealing an interesting property of the adjacency matrix *A*. For instance, one can always relabel the nodes of a tree such that its asymmetric adjacency matrix becomes upper triangular. This immediately follows that the eigenvalues of *A* are all zero. This, however, does not restrict us from investigating the spectrum (distribution of eigenvalues) of river networks as one can always utilize different matrix descriptors of a graph. In particular, here we define a symmetric adjacency matrix *B* which only considers the connectivity of the nodes while ignoring the flow directions, thus:

2 $${b}_{ij}=\{\begin{array}{ll}1: & i\,{\rm{and}}\,j\,{\rm{linked}};\\ 0: & {\rm{otherwise}}\end{array}$$

Using (2), *B* can be written in terms of *A* as *B* = *A* + *A* ^*T*^, where “*T*” represents a transpose operation. Although, *A* cannot be obtained in terms of *B*, in a river network this is possible given that the outlet is known and there is always a unique path from each node to the outlet. Therefore, the flow directions can be ignored and the network can be described with an undirected graph as displayed in the example of Fig. 1c which further can be characterized with a symmetric adjacency matrix shown in Fig. 1d.

The definition of equation (2) directly implies that *B* is a sparse matrix (most elements are zero) with the following properties: (i) *B* is a symmetric matrix, i.e., *b*_*ij*_ = *b*_*ji*_. (ii) The number of 1’s in *i* ^*th*^ row or column is equal to the number of direct neighbors of the *i* ^*th*^ node, i.e., number of links which are directly connected to that node (which is commonly called as degree). Here, we define the number of links connected to each node as the degree of that node. (iii) All the diagonal elements of *B* are zero and therefore the trace (sum of diagonal elements) of *B* is zero. In this work, we focus on the eigenvalue spectrum of the symmetric adjacency matrix *B*.

### Eigenvalue spectrum of modeled channel networks

In this study, we generate river network graphs by using an optimal channel network (OCN) approach which can produce river networks with different branching patterns (representing angle of bifurcation, drainage density etc.^11^). OCNs are in general obtained by finding an optimal topology with a local minimum of the total energy. For each link, the dissipated energy is related to the discharge through an exponent *γ* (*γ* varies between 0 and 1) which is assumed to be constant for the entire network and characterizes the bifurcation pattern (see Methods). The simulated river network using OCN model reproduces several topologic and geomorphic properties of real river networks and has been explored extensively in the recent past^8, 28–33^.

**Figure 2:**
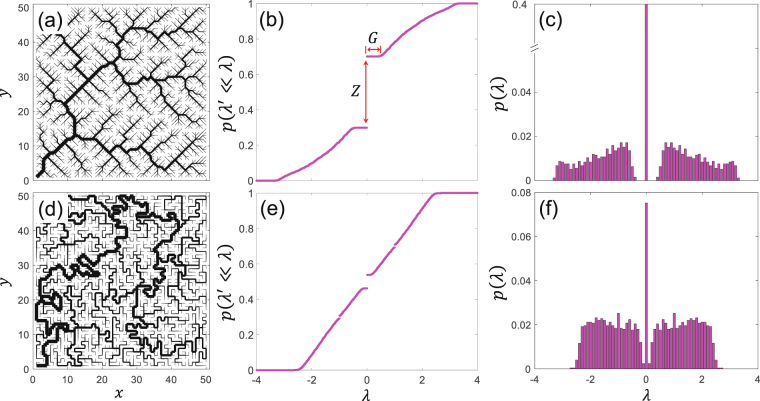
(**a**) A river network generated with an optimal channel network model on a 50 × 50 square lattice. (**b**,**c**) The cumulative density function (cdf) and the probability density function (pdf) of the associated adjacency matrix eigenvalue spectrum. (**d**) A randomly generated spanning tree on the same lattice and (**e**,**f**) its associated cdf and pdf. *G* and *Z* in (**b**) represent spectral gap and nullity, respectively.

Figure 2a depicts an exemplary river network generated via an OCN model while the cumulative probability of its adjacency matrix eigenvalue spectrum is shown in Fig. 2b. As expected, the eigenvalues are symmetrically distributed around the origin. Since eigenvalue distribution is symmetric, the negative part does not carry any new information, thus we can only focus on the positive part of the spectrum. The pdf of eigenvalues is also plotted in Fig. 2c. Figure 2d–f, on the other hand, depict a spanning tree, generated through a random walk on the same lattice, along with its eigenvalue distributions^28^.

Comparing the river network (Fig. 2a) with random network (Fig. 2d), apart from the basic properties listed above which are in common in both networks, one can clearly see three important signatures in the eigenvalue spectra of the river network. The first is the ratio of zero eigenvalues (*N*_*z*_) to the total number (*N*) of eigenvalues *Z* = *N*_*z*_/*N*, which we refer to as nullity. The second is the value of the smallest nonzero eigenvalue that also identifies a forbidden range below which no eigenvalue exists which in the case of the river network of Fig. 2a is found to be ~±0.42 while for the random walk network of Fig. 2d is ~±0.1. We refer to this range as the spectral gap *G*. Note that in the context of spectral graph theory, spectral gap is commonly referred to as the difference between the two largest eigenvalues of the adjacency matrix. Finally, the third property is a significant difference in the shape of pdfs of eigenvalues (see Fig. 2c,f).

In the rest of this manuscript we aim to address how these spectral signatures are related to the geomorphic properties of the stream networks. In particular, we are interested in geometric properties such as the size and shape of the basin, as well as the topology of the river network that leads to a certain branching pattern. The branching pattern of a river network is of significant importance in characterizing a landscape and has been shown to depend on climatic, geologic, biologic and ecologic conditions of the river network landscape^2, 7, 34–36^.

#### Spectral gap and nullity

**Figure 3:**
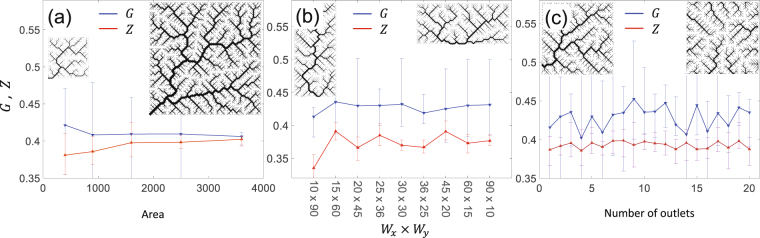
Dependence of the spectral gap *G* and the nullity *Z* vs. (**a**) size, (**b**) shape and (**c**) connectivity of the river networks. In (**a**), all basins are square and the horizontal axis represents the area of the network. In (**b**), rectangular basin shapes with length *W*_*x*_ and width *W*_*y*_ are considered and the horizontal axis represents the aspect ratio. In (**c**), all basins have the same boundary while the number of outlets changes. In all cases blue and red curves show the spectral gap *G* and the nullity *Z*, respectively, while the insets represent examples of river networks at two data points.

In order to investigate the effect of network size, here we generate OCNs of different sizes varying from *N* = 400 to *N* = 3600. To minimize the effect of random initializations of the generated networks, in each case, 10 independent network realization were generated and their ensemble averaged spectral gap and nullity were computed. Figure 3a shows that with increasing size of the network, the spectral gap (*G*) and nullity (*Z*) remain nearly constant despite the fact that the number of eigenvalues is equal to the number of lattice nodes. In a similar manner, the effect of the shape of the river basin can be studied by considering many realizations of drainage networks inside borders with different shapes while all preserving the same drainage area. For this purpose, we consider a rectangular boundary with different width and elongation. As in previous case, the results are reported after ensemble averaging. The spectral gap and nullity are depicted as a function of the basin aspect ratio in Fig. 3b. Similar to the previous scenario, in this case as well the spectral features are barely affected by the elongation of the basin. Finally, a scenario where the river basin can have multiple outlets -a scenario frequently observed in a natural landscape^37, 38^ is considered. A spectral analysis of such networks shows that in this case again the eigenvalue distribution is barely affected by the number of disconnected river networks on the entire landscape (see Fig. 3c). In general, the impact of geometrical properties of a river network on the eigenvalue spectrum of that network is negligible.

**Figure 4:**
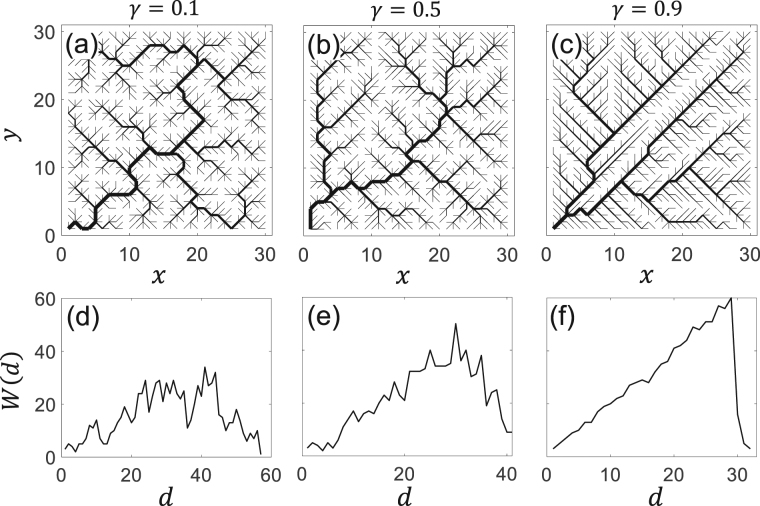
(**a**–**c**) Examples of optimal channel networks generated with *γ* = 0.1, 0.5 and 0.9 respectively. (**d**–**f**) The associated width functions. As can be seen from this figure, with increasing *γ*, the maximum channel length *d*_*max*_ decreases.

We next consider the branching patterns of river networks. As shown by several studies, different external forcing (e.g. climatic, tectonic) can lead to the formation of different drainage branching patterns that may result in different values of *γ* ^11^. In our simulations, different patterns can be produced by varying the energy dissipation exponent *γ*. As illustrated in Fig. 4a–c, by varying *γ* from 0.1 to 0.9 the drainage pattern changes drastically from an intertwisted river network to an entirely straightened pattern. The width functions^11, 28, 39, 40^, characterizing the number of nodes in the network that are located at a distance of *d* from the outlet, associated with these networks are depicted in Fig. 4d–f. According to these figures, the effect of the energy dissipation rate on the bifurcation pattern is well reflected in the shape of the width function. For example, by increasing *γ*, the longest stream length *d*_*max*_ decreases and the peak of the width function shifts from the median stream length towards the maximum value.

**Figure 5:**
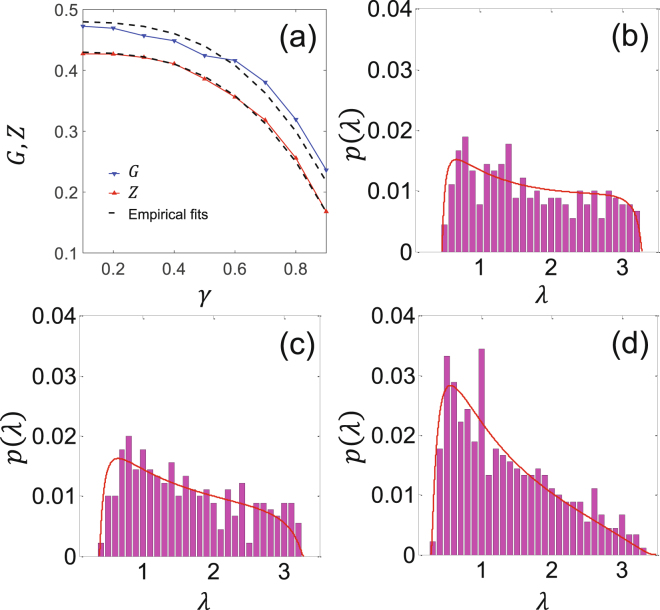
(**a**) Spectral gap *G* and the percentage of zeros *Z* for different energy dissipation exponents *γ*. Each data point in this figure represents an average of 10 realizations of networks under the same conditions. The dashed lines show the empirical fits based on equation (3). (**b**–**d**) The positive part of the eigenvalue distribution associated with river networks shown in Fig. 4(a–c), respectively. The solid red lines in panels show the fitted Johnson’s SB distributions (equation (4)). As can be seen from these pdfs, the asymmetry in the eigenvalue distribution increases with increasing *γ*.

To quantify the effect of different drainage patterns (see Fig. 4) on the eigenvalue spectrum resulting from varying energy exponent *γ*, we compute the spectral gap and nullity as a function of the *γ*. As shown in Fig. 5a, by increasing *γ*, both spectral gap and nullity decrease monotonically. For example, the spectral gap reduces to almost 50% when *γ* increases from 0.1 to 0.9. A further look at the plots of the spectral gap *G* and nullity *Z* (Fig. 5a) versus the energy decay exponent *γ* suggests the following empirical relations:

3 $$G={G}_{0}-{(\gamma /{\gamma }_{0})}^{\tau },\,Z={Z}_{0}-{(\gamma /{\gamma }_{0})}^{\tau },$$

where the two offsets are *G*_0_ = 0.48 and *Z*_0_ = 0.43, while the scale *γ*_0_ and the exponents *τ* (common in both equations) are found to be 1.37 and 3.2, respectively.

#### Distribution of the eigenvalues

The distribution of the eigenvalue spectra of complex networks has been a subject of intensive investigations for the past decade^41^. In particular, it has been shown that the eigenvalue distribution of complex networks do not obey the Wigner’s semicircular distribution which describes the spectra of random symmetric matrices and governs a wide range of disordered systems in physics^41–43^. According to these studies, the eigenvalue spectrum of scale-free networks follow a power-law distribution which itself is rooted in the power-law degree distribution in such networks. In case of river networks generated on a square lattice, on the other hand, the degrees are distributed between 1 to 8. Therefore, of interest would be to compare the distribution of the eigenvalues of simulated river networks with that of well-known networks. As we show in the following, the spectrum of river networks is not governed by the Wigner’s semicircle law^12, 13^, as in random graphs, and neither it follows the power-law distribution of the scale-free networks.

Figure 5b–d depict the distribution of the eigenvalues for the OCNs obtained with different energy decay exponent *γ*, i.e. *γ* = 0.1, 0.5, and 0.9, respectively. Interestingly, in this case, the pdf of eigenvalues changes significantly from an almost uniform toward a skewed distribution where larger eigenvalues are less likely. These pdfs can be approximated with a four-parameter Johnson’s SB distribution, a transformed normal distribution, represented by refs:^44, 45^

4 $$f(x)=\frac{\delta }{\mu \sqrt{(}2\pi )\,z\mathrm{(1}-z)}\,\exp (-\mathrm{0.5(}\alpha +\delta \,\mathrm{ln}(z/(1-z)){)}^{2}).$$

Here, the normalized random variable is defined as $z=\tfrac{x-\zeta }{\mu }$, where *μ* and *ζ* represent scale and mean parameters and *α* and *δ* are two shape parameters (see Fig. 5d–f). Suitable for our purpose, this distribution is defined over the bounded range of *ζ* < *x* < *ζ* + *μ* and can track the skewness of the observed eigenvalue distributions. For example, the skewness index (*α*) of the eigenvalue distribution for the river networks obtained for *γ* = 0.1, 0.5, and 0.9 are *α* = 0.2, 0.35, and 0.8, respectively, whereas for the random network shown in Fig. 2d is ~0.

### Spectrum of experimental river basins

**Figure 6:**
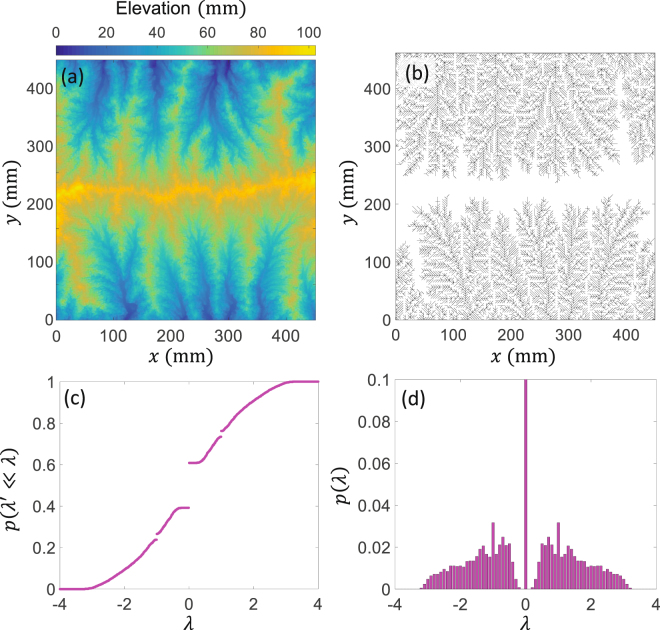
Example of steady state landscape obtained from a physical experiment (see for details^46^). (**a**) The digital elevation map, (**b**) extracted stream network, (**c**) the cumulative density function *p*(*λ*′ ≤ *λ*), and (**d**) the probability density function *p*(*λ*) of the eigenvalues.

To further explore the observed signatures of the eigenvalue distributions, here, we compare our OCN model with physical experiments. Figure 6a shows the digital elevation model (DEM) of the steady state landscape obtained from recently conducted physical experiments on landscape evolution^46, 47^. These experiments were designed to create an evolving landscape under constant uplift rate (20 mm/hr) and rainfall rate (45 mm/hr). The substrate of the eroding material consisted of silica with particle size of *d*_50_ = 25 *μm*. More details about the experimental facility and data collected can be obtained from Singh *et al*.^46^. Figure 6b shows the extracted channel network from the DEM which was used to compute the eigenvalue spectrum. Figure 6c shows the cumulative distribution, whereas Fig. 6d shows the pdf of the eigenvalues for the experimental river network. As can be seen from Fig. 6c, strikingly a similar pattern of eigenvalue spectrum (spectral gap) is observed in the physical landscape suggesting that spectral gap is a distinct signature of river network topology. Future work will focus on exploring how changing external forcing (e.g. climatic, tectonic) can affect the spectrum.

## Discussion and Concluding remarks

In this paper, using a graph-theoretical framework, we investigated the eigenvalue spectrum of river network topology. We utilize river networks simulated through optimal channel network approach as well as river networks extracted from recently conducted physical experiments on landscape evolution. The main results of this study can be summarized as follows:

- The eigenvalue spectrum exhibits striking features including a forbidden range where no eigenvalue exists. We refer to this range as the spectral gap and show that this gap is closely related to the branching pattern of the river network.
- The nullity (number of zero eigenvalues in the spectrum) of the river network adjacency matrix shows similar trend as that of spectral gap.
- Both spectral gap and nullity are independent of the size and shape of the basin as well as the number of outlets. In addition, the eigenvalue pdf is mostly dictated by *γ* (an exponent capturing mechanics of erosional processes). The spectral properties of river networks, therefore, can be seen as representing important characteristics of the bifurcation pattern in river networks.

Our results reveal the potential of spectral graph techniques for investigating river networks’ topology. The proposed spectral features can be utilized as novel geomorphological descriptors of drainage patterns. Such spectral features can characterize a wide range of drainage patterns and go beyond the simple patterns with end and side bifurcation typically described through Horton-Strahler and Tokunaga indices^48, 49^. In addition, the eigenvalue descriptors can be directly extracted from the connectivity matrix of river networks in a single step.

Although we have explored several aspects of the eigenvalues spectrum through a numerical inspection of OCNs, it remains an open problem to derive analytical expressions for some of these findings. In particular, a rigorous derivation of the probability density function of the eigenvalues from the degree distribution would be highly desirable. Finally, of interest would be to explore the properties of real-world river networks in connection with their eigenvalue spectrum.

## Methods

### Optimal channel networks

Optimal channel networks are obtained by locally minimizing the total energy functional which is defined as the sum of energy dissipation in all links of the network, i.e., $E={\sum }_{i=1}^{N-1}\,{L}_{i}{q}_{i}^{\gamma }$. Here, ${L}_{i}{q}_{i}^{\gamma }$ represents the energy dissipated in the *i* ^*th*^ link of the network where *L*_*i*_ and *q*_*i*_ represent its length and discharge respectively^11^. The exponent *γ* is ranged between zero and unity and is an important parameter which characterizes the mechanisms of erosional processes in the stream network^11^. The discharge at the *i* ^*th*^ link can be written in terms of the discharge of upstream links through the adjacency matrix as ${q}_{i}={\sum }_{j}\,{a}_{ij}{q}_{j}+{r}_{i}$, where, the summation is taken over all direct upstream nodes and *r*_*i*_ represents the rainfall rate at the *i* ^*th*^ node. The total energy functional can be simplified by assuming equal distance between each two neighboring points on a lattice which leads to $E={\sum }_{i=1}^{N-1}\,{q}_{i}^{\gamma }$. On the other hand, assuming a uniform precipitation over the entire lattice, i.e., *r*_*i*_ = 1 for *i* = 1, …, *N*, the discharge at the *i* ^*th*^ link is obtained to be ${q}_{i}={\sum }_{k=0}^{N-1}\,{\sum }_{j=1}^{N}\,{a}_{ij}^{(k)}$, where ${a}_{ij}^{(k)}$ represents the matrix elements of *A* ^*k*^. This latter relation is obtained by using a property of the adjacency matrix *A* which states that the number of walks of length *k* from node *i* to node *j* is equal to the entry in the *i* ^*th*^ row and *j* ^*th*^ column of the *k* ^*th*^ power of *A*. By using the discharge relation, the total dissipated energy of the river network can now be written as:

5 $$E=\sum _{i=1}^{N-1}\,{(\sum _{k=0}^{N-1}\sum _{j=1}^{N}{a}_{ij}^{(k)})}^{\gamma }$$

As this relation clearly indicates, for a given energy dissipation rate *γ*, the total energy of the network is solely a function of the adjacency matrix which basically describes the graph topology. In simulations, by generating valid adjacency matrices, we utilize a hill climbing algorithm to find a local minima of the energy functional (equation (5)) and use such optimal topologies as optimal channel networks.

## References

[1] Bertuzzo, E. *et al*. On the space-time evolution of a cholera epidemic. *Water Resour*. *Res*. **44** (2008).

[2] Rodríguez-Iturbe I, Muneepeerakul R, Bertuzzo E, Levin SA, Rinaldo A (2009). River networks as ecological corridors: A complex systems perspective for integrating hydrologic, geomorphologic, and ecologic dynamics. Water Resour. Res..

[3] Zaliapin I, Foufoula-Georgiou E, Ghil M (2010). Transport on river networks: a dynamical approach. J. Geophys. Res..

[4] Rinaldo A, Rigon R, Banavar JR, Maritan A, Rodríguez-Iturbe I (2014). Evolution and selection of river networks: Statics, dynamics, and complexity. Proc. Natl. Acad. Sci..

[5] Czuba JA, Foufoula-Georgiou E (2015). Dynamic connectivity in a fluvial network for identifying hotspots of geomorphic change. Water Resour. Res..

[6] Smith TR, Bretherton FP (1972). Stability and the conservation of mass in drainage basin evolution. Water Resour. Res..

[7] Tucker GE, Slingerland R (1997). Drainage basin responses to climate change. Water Resour. Res..

[8] Molnar P, Ramírez JA (1998). Energy dissipation theories and optimal channel characteristics of river networks. Water Resour. Res..

[9] Benda L (2004). The network dynamics hypothesis: How channel networks structure riverine habitats. BioScience.

[10] Barabasi A-L, Albert R (1999). Emergence of scaling in random networks. Sci..

[11] Rodríguez-Iturbe, I. & Rinaldo, A. Fractal river basins: Chance and self-organization. *Camb. Univ. Press. New York* (2001).

[12] Albert, R. & Barabasi, A.-L. Statistical mechanics of complex networks. *Rev*. *Mod*. *Phys*. **74** (2002).

[13] Boccaletti S, Latora V, Moreno Y, Chavez M, Hwang DU (2006). Complex networks: Structure and dynamics. Phys. Rep..

[14] Horton RE (1932). Drainage-basin characteristics. Eos, Transactions Am. Geophys. Union.

[15] Horton RE (1945). Erosional development of streams and their drainage basins; hydrophysical approach to quantitative morphology. Geol. Soc. Am. Bull.

[16] Shreve RL (1966). Statistical law of stream numbers. J. Geol..

[17] Shreve RL (1966). Stream lengths and basin areas in topologically random channel networks. J. Geol..

[18] Shreve RL (1967). Infinite topologically random channel networks. J. Geol..

[19] Chung, F. R. Spectral graph theory. *CBMS Reg*. *Conf*. *Ser*. *Math*. *Am*. *Math*. *Soc*. **92** (2011).

[20] Cvetkovic, D. M. & Rowlinson, P. Spectral graph theory. *Top*. *algebraic graph theory*, eds Beinke, L. W. & Wilson, R. J., Camb. Univ. Press. 88–112 (2004).

[21] Gutman, I. Chemical graph theory - the mathematical connection. *Adv*. *Quantum Chem*. **51**, 125–138 (2006).

[22] Van Mieghem, P. Performance analysis of communications networks and systems. *Camb. Univ. Press. Camb*.

[23] Spielman, D. A. Spectral graph theory and its applications. *48th Annu*. *IEEE Symp*. *on Foundations Comput*. *Sci*. IEEE, 29–38 (2007).

[24] Mohar, B. & Poljak, S. Eigenvalues in combinatorial optimization. *Comb*. *Graph*-*Theoretical Probl*. *Linear Algebr*. (eds Brualdi, R., Friedland, S. & Klee, V.) Springer-Verlag, New York **50**, 107–151 (1993).

[25] Scheidegger AE (1967). On the topology of river nets. Water Resour. Res..

[26] Tejedor A, Longjas A, Zaliapin I, Foufoula-Georgiou E (2015). Delta channel networks: 1. A graph-theoretic approach for studying connectivity and steady state transport on deltaic surfaces. Water Resour. Res..

[27] Tejedor A, Longjas A, Zaliapin I, Foufoula-Georgiou E (2015). Delta channel networks: 2. metrics of topologic and dynamic complexity for delta comparison, physical inference, and vulnerability assessment. Water Resour. Res..

[28] Abed-Elmdoust A, Miri M, Singh A (2016). Reorganization of river networks under changing spatio-temporal precipitation patterns: an optimal channel network approach. Water Resour. Res..

[29] Rinaldo A (1992). Minimum energy and fractal structures of drainage networks. Water Resour. Res..

[30] Rodríguez-Iturbe I (1992). Energy dissipation, runoff production, and the three-dimensional structure of river basins. Water Resour. Res..

[31] Rigon R, Rinaldo A, Rodríguez-Iturbe I, Bras RL, Ijjász-Vásquez E (1993). ptimal channel networks: A framework for the study of river basin morphology. Water Resour. Res..

[32] Rinaldo A, Rodríguez-Iturbe I, Bras RL, Ijjász-Vásquez E (1993). Self-organized fractal river networks. Phys. Rev. Lett..

[33] Paik K, Kumar P (2008). Emergence of self-similar tree network organization. Complex..

[34] Rinaldo A, Dietrich WE, Rigon R, Vogel G, Rodríguez-Iturbe I (1995). Geomorphological signatures of varying climate. Nat..

[35] Tucker GE (2004). Drainage basin sensitivity to tectonic and climatic forcing: Implications of a stochastic model for the role of entrainment and erosion thresholds. Earth Surf. Process. Landforms.

[36] Hooshyar, M., Singh, A. & Wang, D. Hydrologic controls on junction angle of river networks. *Water Resour*. *Res*. **53**, doi:10.1002/2016WR020267 (2017).

[37] Bonnet S (2009). Shrinking and splitting of drainage basins in orogenic landscapes from the migration of the main drainage divide. Nat. Geosci..

[38] Goren L, Willett SD, Herman F, Braun J (2014). Coupled numerical-analytical approach to landscape evolution modeling. Earth Surf. Process. Landforms.

[39] Naden PS (1992). Spatial variability in flood estimation for large catchments: The exploitation of channel network structure. Hydrol. Sci. J..

[40] Snell JD, Sivapalan M (1994). On geomorphological dispersion in natural catchments and the geomorphological unit hydrograph. Water Resour. Res..

[41] Dorogovtsev, S. N., Goltsev, A. V., Mendes, J. F. F. & Samukhin, A. N. Spectral of complex networks. *Phys*. *Rev*. *E*. **68** (2003). 10.1103/PhysRevE.68.046109 14683004

[42] Farkas, I. J., Derenyi, I., Barabasi, A. L. & Vicsek, T. Spectra of “real-world” graphs: Beyond the semicircle law. *Phys*. *Rev*. *E*. **64** (2001). 10.1103/PhysRevE.64.026704 11497741

[43] Chung F, Lu L, Vu V (2003). Spectra of random graphs with given expected degrees. Proc. Natl. Acad. Sci..

[44] Johnson NL (1949). Systems of frequency curves generated by methods of translation. Biom..

[45] Siekierski K (1992). Comparison and evaluation of three methods of estimation of the johnson sb distribution. Biometr. Jour..

[46] Singh, A., Reinhardt, L. & Foufoula-Georgiou, E. Landscape reorganization under changing climatic forcing: Results from an experimental landscape. *Water Resour*. *Res*. **51**, doi:10.1002/2015WR017161 (2015).

[47] Tejedor, A., Singh, A., Zaliapin, I., Densmore, A. & Foufoula-Georgiou, E. Scale-dependent erosional patterns in steady and transient state landscapes. *In Revis*. 10.1126/sciadv.1701683 PMC5617378 28959728

[48] Peckham SD (1995). New results for self-similar trees with applications to river networks. Water Resour. Res..

[49] Peckham, S. Self-similarity in the three-dimensional geometry and dynamics of large river basins. *PhD thesis*, *Univ*. *Color*., *Boulder*, *Colo*. (1995b).

